# The two-brain approach: how can mutually interacting brains teach us something about social interaction?

**DOI:** 10.3389/fnhum.2012.00215

**Published:** 2012-07-24

**Authors:** Ivana Konvalinka, Andreas Roepstorff

**Affiliations:** Center of Functionally Integrative Neuroscience, Aarhus UniversityAarhus, Denmark

**Keywords:** dual EEG, hyperscanning, social interaction, interpersonal analysis, decoding

## Abstract

Measuring brain activity simultaneously from two people interacting is intuitively appealing if one is interested in putative neural markers of social interaction. However, given the complex nature of interactions, it has proven difficult to carry out two-person brain imaging experiments in a methodologically feasible and conceptually relevant way. Only a small number of recent studies have put this into practice, using fMRI, EEG, or NIRS. Here, we review two main two-brain methodological approaches, each with two conceptual strategies. The first group has employed two-brain fMRI recordings, studying (1) turn-based interactions on the order of seconds, or (2) pseudo-interactive scenarios, where only one person is scanned at a time, investigating the flow of information between brains. The second group of studies has recorded dual EEG/NIRS from two people interacting, in (1) face-to-face turn-based interactions, investigating functional connectivity between theory-of-mind regions of interacting partners, or in (2) continuous mutual interactions on millisecond timescales, to measure coupling between the activity in one person's brain and the activity in the other's brain. We discuss the questions these approaches have addressed, and consider scenarios when simultaneous two-brain recordings are needed. Furthermore, we suggest that (1) quantification of inter-personal neural effects via measures of emergence, and (2) multivariate decoding models that generalize source-specific features of interaction, may provide novel tools to study brains in interaction. This may allow for a better understanding of social cognition as both representation and participation.

## Introduction

Much of previous work in social cognition has investigated behavior and brain activity of individuals in isolation, while immersed in a social context. This approach has had some obvious shortcomings, the main criticism being that the studied social contexts have not involved actual interactions with another person (Sebanz et al., 2006a; Schilbach, 2010). Such scenarios have thus not facilitated a mutual exchange of information, much less one that takes place continuously in real-time. Recent approaches have aimed at filling this gap by quantifying behavioral and neural underpinnings of social interactions engaging two or more people. However, these studies have generally neglected the inter-personal (between-person) dynamics of the interaction, by focusing on the intra-personal (within-person) effects.

Is there something fundamental missing when we only focus on the intra-personal effects? If part of the social signature lies in the inter-personal aspect of the interaction, we may be overlooking some key effects of our experiment by ignoring this. Previous behavioral studies have demonstrated emergent, stable patterns of interaction when looking at inter-personal entrainment between people in scenarios involving rhythmic behavior, i.e., rocking in chairs, swinging pendulums, finger-tapping (Schmidt et al., 1998; Richardson et al., 2007; Konvalinka et al., 2010). They have helped to better understand the mechanisms underlying continuous interactions, capturing the real-time aspect most real life interactions contain.

Recent interaction experiments have also begun to investigate inter-brain processes, in order to understand what goes on in two brains as they interact (Dumas, 2011). The approach of measuring activity from two brains simultaneously, using fMRI, EEG, or more recently NIRS, known as hyperscanning (Montague et al., 2002), has only been around for one decade, because of the complex set-up and quantification of between-brain effects, which require careful planning and application of new methods. Given that these technologies are available, are we now tackling the right questions?

In this paper, we show how the field has gone from studying individuals towards a two-person social neuroscience, and furthermore towards a two-brain science. We review two main groups of two-brain studies: (1) fMRI studies that have employed (a) turn-based interactions on a timescale of seconds or (b) pseudo-interactive settings, scanning one person at a time, and (2) dual EEG/NIRS studies that have employed (i) face-to-face turn-based interactions, or (ii) mutually interactive settings on a millisecond timescale. We discuss the questions these various approaches have addressed.

While studying two interacting brains seems to be an important future step to the study of social cognition, we feel that there is a real need to consider what experimental designs and analysis approaches should be implemented to take advantage of this approach. The difficulties in quantifying inter-brain effects of interactions may thus not be primarily due to lack of methods, but due to not knowing what question to ask. The real question is, what can we learn about social interaction from two interacting brains that we cannot learn from individual brains immersed in an interaction? We discuss future perspectives and approaches, and propose that an informational, machine-learning approach to two-brain studies may be beneficial in disentangling inter-personal neural processes.

## Isolated minds versus interacting minds—two accounts of social cognition

Two main conceptual approaches have been taken to study the mechanisms of social cognition. The first has been adapted from a representationalist perspective (see Fuchs and De Jaegher, 2009 for further discussion), considering social cognition to be a process that goes on within an individual who creates models of other people's mental states and incorporates them with his/her own. The underlying hypothesis of this approach is that processes enabling us to socially interact with other people are entirely internalized, and can be understood by studying individual minds. This perspective is supported by the conjecture proposed by Brothers (1990), that there is a set of brain regions dedicated to social cognition, which comprise the “social brain” (see Adolphs, 1999; Frith, 2007 for reviews).

Previous research in social cognition has extensively adopted this view, by placing human participants in MR scanners, and having them respond to “social” stimuli by observing pictures or videos of others, rating untrustworthy faces, making decisions whether to trust a co-player in an economic game, and so on. These experiments have been thought to involve processes engaged in understanding other people via representation of their minds or mental states (Lieberman, 2007). They have identified key brain areas, which have been thought to comprise the social brain: the amygdala, orbital frontal cortex (OFC), temporal cortex, medial prefrontal cortex (MPFC), adjacent paracingulate cortex, and the “mirror neuron system (MNS)” (Frith, 2007). While these brain areas have consistently “lit-up” in participants engaged in isolated social experiments, each has been shown to have many functions, including those that are not necessarily involved in the processing of social information. One example is the amygdala, which has been shown to activate during processing of fearful faces, untrustworthy faces, as well as stimuli that are considered to be both positively and negatively valuable, even when they are not social (see Frith, 2007).

More crucially, these studies have mainly explored social cognition from the point of view of the observer. It thus remains largely unexplored how these identified brain regions make everyday online interactions with others possible, involving real-time coordination of actions, goals, and intentions. After all, social interaction is a largely dynamic process, which is about much more than observing and imitating.

This “isolated brain” approach has been criticized, as the social contexts studied have not immersed the participant in a true interaction with another person, allowing a mutual exchange of information and hence a mutual coordination of actions. The idea behind this is that social cognition is fundamentally different when an individual is actively engaged in an interaction, rather than a mere observer (De Jaegher, 2009; Schilbach, 2010). Specifically, the former approach does not explain how perception, action, and cognition are modulated during real-time interactions with other people. For example, the mechanisms underlying temporal aspects of coordination and joint decision making seem poorly understood so far. These mechanisms cannot be explained merely by looking into brain activations of individuals, but require experimental set-ups involving person–person interactions, and analysis methods that quantify inter-brain interactions.

These criticisms have lead to a second approach, which considers social cognition to be a process that goes on between two or more people while interacting, “as they coordinate their actions in space and time to bring about a change in the environment” (Sebanz et al., 2006a). This “joint action” or “interactive” approach has thus moved away from studying minds in isolation and towards studying minds in interaction.

However, while immersing people in two-way interactions, this approach has still mostly quantified individual, intra-personal processes of each coordinating partner. For example, many interactive studies have measured brain activity (and/or behavior) of only one individual, while in an interaction with another, non-scanned partner (examples of brain studies include Sebanz et al., 2007; Redcay et al., 2010). While these studies are indeed interactive, we do not review them here as they have only measured intra-brain processes.

## When interactions matter

It has been proposed that in order to take advantage of the interactive approach, the field needs to move toward quantifying the inter-personal co-regulated coupling between interacting partners, while they mutually and continuously affect one another (De Jaegher et al., 2010). When we interact with another person, our brains and bodies are no longer isolated, but immersed in an environment with the other person, in which we become a coupled unit through a continuous moment-to-moment mutual adaptation of our own actions and the actions of the other (Konvalinka et al., 2010). This dynamical interactive process has been shown to result in an alignment of behavior (Richardson et al., 2007; Schmidt and Richardson, 2008; Konvalinka et al., 2010), posture (Shockley et al., 2003), autonomic systems (Muller and Lindenberger, 2011) such as respiration (McFarland, 2001) and cardiac rhythms (Konvalinka et al., 2011), and potentially neural rhythms (Dumas et al., 2011; Hasson et al., 2012) between the two individuals. These inter-personal couplings across modalities appear to create bonds that facilitate successful interactions, and might then be crucial in identifying mechanisms underlying continuous social interactions.

It is important to note that the studies employing the isolated brain approach are still fundamental, as they have laid the groundwork for the understanding of social cognition, and have consistently identified the same key brain areas used when engaging in an interaction. However, to advance the field further, it is critical to identify when interaction studies are necessary, and what may be gained from them.

The enactive account of cognition argues that social understanding comes from the dynamical process whereby people become a coupled unit through the moment-to-moment interaction, which cannot always be disentangled into separate autonomous entities, and is hence emergent (Fuchs and De Jaegher, 2009; De Jaegher et al., 2010; Dumas, 2011). Taking a conversation between two people as an example, it would be difficult to make sense of the interaction if the two interlocutors were analyzed separately. While we know which person the speech at each instance belongs to, the conversation only begins to make sense when we analyze it as a whole of two interlocutors' speeches. If we want to capture the interaction dynamics, we must thus treat the interacting members as a coupled unit. Here we give two experimental examples of situations when this becomes crucial.

In a previous study of ours, we carried out a joint finger tapping experiment, in which we aimed to quantify the ongoing dynamics between two interacting participants (Konvalinka et al., 2010). The participants were asked to synchronize with their auditory feedback, which either came from their own tapping, the other person's tapping, or the computer metronome, thus manipulating the degree of interaction. We found a stable, emergent pattern of interaction when the two members could both hear each other. Their inter-tap intervals (ITIs) oscillated on a tap-to-tap basis, such that if one went faster on the last tap, the other would speed up on the next one and the one would simultaneously slow down. This pattern was quantified using windowed cross-correlations, showing continuous lag –1 and lag +1 coefficients, and a negative lag 0 cross-correlation – hence, two mutual, continuous followers of each other's previous tap. Moreover, synchronization analysis showed that the participants were just as good at synchronizing with the variable, adaptive other as with the unvarying, non-adaptive computer. However, they were worse when their partner was both unpredictable and non-responsive (one-way coupling).

This inter-personal analysis provided a way of quantifying the ongoing, stable patterns of the mutual interaction. We were able to show that when two people engage in a synchronization task, they do better when they both continuously and mutually adapt to one another's actions—in other words, when they become two followers, instead of adopting a leader-follower dynamic. The stability of the two-way interaction without a distinct leader and follower has also been found in studies of movement improvisation (Noy et al., 2011).

Another study from our group examined the role of linguistic alignment in joint decision-making during a low-level perceptual task (Fusaroli et al., 2012). In the original study by Bahrami et al. (2010), the two members in each pair were presented with visual displays containing a dim target, and asked to decide individually which part of the screen the target appeared on. Subsequently, the dyads were asked to share their decisions with each other, and make a joint decision about the location of the target if they previously disagreed. When the participants had similar visual sensitivities, two heads performed better than the best individual one. The Fusaroli et al. study showed that the relative success of the dyads correlated with how well they were able to establish a common language for their metacognition. In other words, the better the dyads were at aligning their linguistic practices and vocabularies over time, the better their task performance. This inter-personal analysis thus shows that the dynamic characteristic of linguistic interaction has an important role in social coordination and joint decision-making.

These two examples show that inter-personal effects can be key in identifying patterns of interaction, both on a low-level of entraining motor systems and on a higher-level of perceptual decision-making. Therefore, important interaction patterns, which are relevant to the ongoing interaction, can be overlooked if the dyads are not studied as a coupled unit.

The “isolated brain” and “interaction” approaches therefore seek out to explore very different mechanisms. The isolated brain approach taps into individual social processes, engaged during observation of other people's actions, representation of other people's mental states, and sometimes more basic perceptual and motor processes, which may or may not be related to social processes.

The interactive approach explores underlying mechanisms needed to engage in an interaction with another person, such as mutual coordination and cooperation. These include both intra- and inter-personal processes, and can be either representational or dynamical mechanisms. Both perspectives have been adopted in two-brain studies, as reviewed in the following two sections, and they complement each other in quantifying different time-scales and properties of interactions.

## Two-brain approaches using fMRI

Two-person interactions have scarcely been employed in studies measuring neural activity, particularly those that measure brain activity from both interacting members at the same time. In the case of fMRI, such studies require each person to lie still in the scanner and yet be able to interact with another person. This is only possible through a computer interface, which induces problems of ecological validity as well as time-delays, making interpersonal cooperation and coordination difficult (King-Casas et al., 2005). Simultaneous recordings of brain activity complicate these problems even further.

But is it just a matter of finding the right experimental paradigm and overcoming the methodological constraints that poses a problem? What is it that we hope to find by looking into two interacting brains? On an abstract level, we might think about looking for a signature of shared representations of intentions, goals, and actions (Sebanz et al., 2006b; Anders et al., 2011). However, in neural terms, it is unclear how shared representations would be anatomically and/or temporally represented. Social interaction is a highly complex process, engaging numerous networks in the brain, and time-scales ranging from milliseconds to minutes, hours, even years (Hari et al., 2010). Therefore, it becomes difficult to first hypothesize about, and even more to quantify these different brain networks and time scales, which give rise to and modulate ongoing social interactions.

### Turn-based interactions

Only in the last decade have these problems been addressed empirically. Montague et al. (2002) were the first to study interactions using hyperscanning, by measuring fMRI from two brains at the same time. They used a simple deception task, where the “sender” was presented with a red or green screen, and transmitted red or green to the “receiver”, who in turn had to determine whether the “sender” communicated the truth about what he/she saw. The receiver was given a reward if he/she guessed correctly; otherwise, the sender received a reward. Coherence between brains was found at 0.04 Hz, which corresponded to the base frequency of the game. A cluster of activity was identified in the supplementary motor area (SMA) of both brains, but was stronger in the brain of the sender. While this study opened the doors to research of simultaneous brain recordings during interactions, showing that this is indeed possible, it involved significant time delays between stimulus and response (Hari and Kujala, 2009).

Other two-brain experiments involving fMRI defined this two-brain interaction in the context of an information transfer between the brains of senders and perceivers. King-Casas et al. (2005) used an economic trust game to show, using the hyperscanning procedure, that reciprocity in one player predicts the future trust in the other interacting player. The study found that response magnitude in the caudate nucleus correlated with the ‘intention to trust’. Moreover, as reputations developed, there was a temporal transfer of the “intention to trust” between the two players' brains.

Another fMRI study employing the hyperscanning approach was carried out by Saito et al. (2010). In a study of joint attention, pairs of participants were scanned while engaging in a real-time gaze exchange. The setup consisted of infrared eye-tracking systems and video cameras, enabling live video images of each respective partner's eyes and eyebrows. The task in concordant runs was to look at a cued target presented below the partner's eyes, either following the target cue as it changed color, or the partner's gaze towards the target. In discordant runs, the task was to look at the opposite side to the cued target. Inter-personal correlation analysis of residual time-courses revealed higher correlations in the right inferior frontal gyrus, an area thought to be part of the MNS, in paired participants compared to non-paired participants.

The study by Montague et al. was the first to begin to explore brain-to-brain interactions between two people, employing “joint action” settings, with timescales of interaction on the order of seconds. The study was innovative and successful in correlating social processes with individual brain activity, as well as information exchange between brains, corresponding to the game base frequency. However, much the same as the second study, the timescale did not capture the moment-to-moment interactions of two mutually coordinated individuals.

To clarify this point further, we compare this behavioral exchange to that of two people sending text messages back and forth to one another. One has to wait to receive the message from the other before responding. This joint action scenario does not capture the automatic and more immediate influence of mutual information exchange on the dyad's actions (such as in face-to-face interactions), but does capture how transmitted information and inferred mental states are represented in the two interacting members.

The third study employed a real-time interaction, involving mutual gaze between participants. This paradigm was novel as it involved a bidirectional real-time exchange of gaze, and will likely be extended to future studies of joint attention. It also took advantage of the hyperscanning technique, showing higher similarities in brain activity between real pairs compared to surrogate pairs. However, given the limited physical construct of fMRI, the task had to be constrained to a limited, less dynamic, exchange of gaze.

### Pseudo-interactive studies

Other fMRI studies investigated the flow of information between two partners' brains, without the use of hyperscanning, but by scanning the two partners one after another during offline interactions. One study looked at pairs engaged in a game of charades, where the sender gestured words to the perceiver. The study showed that the activity in the sender's brain proceeded activity in the perceiver's brain (Schippers et al., 2010). Moreover, this activity was found in brain areas thought to be involved in mentalizing and mirroring. The second study investigated flow of affective information between two people engaged in a facial communication (Anders et al., 2011). Similarly, the study reported that activity in the brain of the “sender” predicted the activity in the brain of the “receiver” with a temporal delay. The third study looked at brain coupling between speakers' and listeners' brains, reporting temporally coupled brain activity between the speakers and listeners, which diminished in the absence of communication (Stephens et al., 2010). These experiments employed one-way interactions, as participants were either shown videos of each other's gestures or facial expressions, or communicated/received a speech, offline.

The studies in this section have identified some of the same brain areas found with the “isolated brain” approach, such as those implicated in mirroring and mentalizing. They all take a representational approach to study two interacting brains, by investigating how other people's mental states are represented in the brain of the observer/receiver of information.

By scanning one participant a time, while treating the two brains as a coupled unit, these studies investigated information transfer from one brain to another. Schippers et al. and Stephens et al. used between-brain Granger-causality analysis and between-brain correlation analysis, respectively, and the study by Anders et al. employed between-brain multivariate pattern recognition analysis. All three studies compared social conditions to non-social conditions, testing well-defined hypotheses about the neural mechanisms underlying information transfer between brains. This approach directly extends previous findings of “isolated brain” studies, carried out in the absence of interaction, to situations of unidirectional interactions where one person receives a message from another.

However, as one-way interactions do not rely on an ongoing two-person exchange of information, this approach cannot capture the mutual influence of the interaction. The receiver of information is the main subject of investigation, hence this approach relies on a first-person representation of mental states (Schilbach, 2010). One person (the receiver) tunes into the brain state of another, while the other (the sender) is in the absence of an interaction.

In summary, these two-person fMRI studies seem to successfully operate without hyperscanning. Avoiding the complicated set-up requiring synchronization of two fMRI scanners, the pseudo-interactive settings quantify two-brain effects of unidirectional interactions, while still maintaining the brain-to-behavior synchronization between the two participants.

### Is hyperscanning necessary for two-brain science?

What has been gained by scanning two people at the same time? While the studies in section “Pseudo-interactive Studies” did not require the use of hyperscanning, the studies by Montague et al. and King-Casas et al. were designed to take advantage of its use. If we take the former study into account, even if the roles of sender/receiver had been simulated, or used as in a pseudo-interactive setting, the scanned participant might not have behaved in the same way as the simulation used for the other participant. Whether the same coherence between brains would have been found in a pseudo-interactive setting, is an interesting question in its own right, and could be addressed by scanning the same participants again, one after another (i.e., playing two rounds of the game). This could answer the question regarding how much of this coherence is related to the interactive setting the participants are in.

The study by Saito et al. identified inter-brain correlations in areas belonging to the MNS, arguing that these regions are involved in the sharing of intention during eye contact. The same line of thought from the previous paragraph applies to this study. Moreover, the MNS has been quite successfully studied in individual brains, which again brings us back to the question of how to go beyond mirroring and observing and towards participating and interacting when employing two-brain settings.

More importantly, the question remains whether we learn more about mechanisms of social interaction by simultaneously measuring brain activity from both people interacting, than by either measuring activity from (i) only one person, engaged in an interaction with another, or (ii) two people separately, engaged in a more controlled, one-directional interaction. The answer to that, we argue, depends on what we aim to find. If we are interested in (1) intra-personal effects of people engaged in an interaction, including representations of other people's actions and mental states, or (2) informational flow between designated senders and receivers, then there is no benefit to hyperscanning. However, if we aim to find inter-brain effects that emerge from the mutual interaction, then it is important to hyperscan—given that there are such inter-personal effects, and that they are not merely related to the similarity in behavior.

These inter-personal effects are easier to conceptualize on a behavioral than a brain level, as two people can directly become coupled through their behavior. The brain-to-brain coupling concept has been proposed to emerge when two brains are immersed in an interaction, with the environment as a passive conductor through which signals pass, coupling the brains together (Hasson et al., 2012). We would phrase this as the following: the moment-to-moment interactions between two brains can so far be understood as a two-way behavioral stimulus-to-brain coupling, such that the behavior of one person is coupled to the brain of the other, and in turn the behavior of the other is coupled to the brain of the one. In effect, the interaction thus becomes an action-perception loop within and between two individuals (Hari and Kujala, 2009). In addition, there might be a brain-to-brain coupling mechanism that does not directly follow from behavioral coupling, but is a result of inter-individual top-down modulations during interaction (we have previously described this as top-top interactions, see Roepstorff and Frith, 2004).

These mechanisms have been predominantly studied using electrophysiological techniques. EEG has become popular for interaction studies involving timing in interpersonal coordination, given its superior temporal resolution over fMRI, its less interfering construct, and its considerably reduced time lags between systems. This is an advantage for studies of social interaction, as these techniques are able to capture short time scales that operate at the level of natural face-to-face interactions. As a result, dual-EEG studies have become increasingly trendy in the last four years. In the next two sections, we briefly review the studies and findings to date.

## Two-brain studies using dual EEG/NIRS recordings

### Turn-based face-to-face interactions

The first group to simultaneously record EEG from two or more interacting members was Babiloni et al. (2006), during a 4-person card game. The game is played with two pairs of players, those situated north and south against those at west and east. The cards are played in a clockwise order, starting with the player to the dealer's left. The remaining players are asked to play a card of the leading suit if they have one, otherwise a card of another suit. The highest card of the leading suit wins. The authors computed partial directed coherence [a Granger-causality approach in the frequency domain (Baccala and Sameshima, 2001)] between selected regions of interest of different pairs of brains, as a measure of inter-brain functional connectivity. The study reported directed coherence between activity in the ACC in the brain of the player who begins the round (i.e., the leader) and activity in the right prefrontal and parietal areas of the leader's partner. These causal links between prefrontal areas of participants were reported in the beta frequency band (but are reported to be representative of results in other frequency bands).

Similar studies from the same group followed, further probing into decision-making during interactive games, and refining the technique (Babiloni et al., 2007a,b; Astolfi et al., 2010a,b; De Vico Fallani et al., 2010). By contrasting patterns from different pairs of participants in the same card game paradigm, one study reported that only members belonging to the same team showed significant functional connectivity in the alpha, beta, and gamma frequency bands (Astolfi et al., 2010b). Moreover, the functional connectivity findings suggested a causal relation between signals estimated to be in the prefrontal areas of the leader and signals from the ACC and parietal areas of the leader's partner. These findings are notably different from those reported in the previous study, which found correlated activity between the leader's ACC and partner's prefrontal/parietal areas. One explanation for this could be a difference in strategies between the leaders/partners (whose roles may be swapped) in the two experiments. It could be that the leaders from the first study were more actively engaged in figuring out their partner's strategy (i.e., representing their partner's intentions), or more effortful in deciding which card to play, similar to the partners in the second study. This is merely speculation, but it does show the importance of quantifying neural processes underlying moment-to-moment interactions between players, as opposed to pooling over long epochs, which contain changes in strategies and outcomes (as discussed in Hari and Kujala, 2009).

Another study from the group measured multi-person EEG in an Iterated Prisoner's Dilemma experiment (De Vico Fallani et al., 2010). They used Granger-causality and graph theory to try and identify a connectivity pattern between brains, which allowed them to predict which pairs adopted a non-cooperative strategy. The only two pairs that consisted of two defectors each had significantly less inter-brain connectivity as well as higher modularity than pairs who adopted other strategies (i.e., cooperative, tit-for-tat, mixed). A recent NIRS-based hyperscanning study of a cooperation-competition game also showed coherence between brains during cooperation, but not competition, which could not be explained merely by the similarities in action (Cui et al., 2012).

These studies have situated pairs of participants in interactive settings, employing economic game approaches. This has allowed for investigation of inter-personal processes underlying two-way neural interactions on a millisecond timescale, as captured using multi-EEG recordings. Utilizing previous findings from the “isolated brain approach”, these studies have used anatomical representations employed in decision-making, to define regions of interest. They have shown correlated brain activity between two people when they cooperate, which diminishes when they compete or defect. However, the behavioral coupling between individuals did not take place on a millisecond timescale, but was rather a turn-based communication. One difference between these studies and those described in the previous section (“Turn-based interactions”) is that the interactions took place face-to-face, and not via an interface, hence situating the participants in a more natural setting.

Given that the pairs in these studies were not designated roles of “senders” and “receivers,” but were in a more natural interaction with one another, the studies could not have been carried out without the use of simultaneous brain recordings. The reason for this is that the players' strategies could not have been predicted beforehand, and hence could not have been simulated in a setting employing a unidirectional interaction. Moreover, the studies found significant brain connectivity patterns only between players that were part of the same team, which is a unique finding showing that these neural similarities are not only a result of the similarity in sensorimotor feedback, and thus cannot be simulated by replacing the players with computers.

### Studies of mutual, ongoing interactions

Other groups employing simultaneous EEG recordings have investigated scenarios of ongoing interpersonal coordination, probing into social coordination dynamics. The first of such studies was carried out by Tognoli et al. (2007), who recorded dual EEG on pairs that were asked to produce self-paced rhythmic finger movements, with or without visual feedback of each other's hand. EEG time-frequency analysis revealed a pair of oscillatory components over the right centro-parietal cortex—named phi1 and phi2, making up the phi complex—in the 9–12 Hz frequency range, which were associated with participants' independent and synchronized movements, respectively. One was increased when participants produced independent movements, and the other was enhanced during coordinated behavior. These components were suggested to belong to the human MNS, hence inhibiting and enhancing the MNS. Despite the simultaneous EEG recordings, however, this study did not look at inter-brain interactions between interacting partners.

A different approach was taken by Lindenberger et al. (2009), who looked at inter-brain phase synchronization. They recorded dual EEG while pairs of guitarists played a short melody together. They found phase synchronized theta and delta oscillations both within and between brains prior to and while playing the melody together. As the authors discuss, given that the reported rhythms were all in the low EEG frequency range, one plausible explanation could be that the similarities in sensorimotor feedback (at least partially) contributed to the inter-brain synchronization.

Another dual EEG study that looked at inter-brain phase synchronization during a real-time, continuous interaction was carried out by Dumas et al. (2010). They used a continuous, mutual hand imitation task, in which the participants were asked to spontaneously imitate each other's hand movements when the felt like it, in one task; or, in another task, one participant was asked to imitate the hand gestures of the other member (i.e., follow), while the other was asked to generate own hand gestures (i.e., lead). The interacting partners were visually coupled, able to see each other's hands through a double video system. The authors looked at the phase locking value for each pair of electrodes between the two brains, computing phase synchronization between brains in various frequency bands. They found inter-brain synchronization between behaviorally synchronized versus non-synchronized episodes in alpha-mu, beta, and gamma frequency bands between the right centro-parietal, central and right parieto-occipital, and centro-parietal and parieto-occipital regions, respectively, but no differences between the imitative versus non-imitative conditions. The study showed that in an ongoing mutual interaction, inter-brain oscillatory couplings accompany behavioral synchrony and turn taking.

Finally, a study by Dodel et al. (2011) measured dual EEG from two-member expert and novice teams performing a simulated combat scenario, to investigate brain signatures of team performance. By computing local subspaces of joint brain dynamics, the study found that novice teams had a higher intrinsic dimensionality than expert teams. Moreover, the study identified a signature specific to team coordination, by contrasting true teams to surrogate teams.

While situating participants in real-time, millisecond level interactions, the two-brain studies described in this section have quantified inter-personal neural processes underlying ongoing social coordination. The studies have thus benefited from having data from two simultaneously interacting brains. Moreover, they have begun to define new experimental paradigms and analyses within the two-person dynamical systems framework.

Taking their lead from the enactive approach to social cognition, the studies have explored modulations of brain rhythm amplitudes and coupling between the brain activities of interacting partners during tasks of mutual ongoing coordination. They have found intra-personal modulation of amplitudes in the 9–12 Hz frequency range during coordination of actions. Moreover, they have consistently reported phase synchronization between prefrontal and centro-parietal brain areas of interacting partners, as well as potential signatures of interpersonal coordination.

These inter-brain phase synchronies have been found across a wide range of frequencies, including delta, theta, alpha, beta, and gamma. These frequencies likely also correspond to a wide range of cognitive and/or interactive processes. Time scales corresponding to perception, cognition, and action, have been shown to range from less than 1 ms to hundreds of milliseconds for stimuli and brain processes (Hari and Kujala, 2009). For example, another person's actions can be predicted 100–150 ms beforehand. Moreover, cortical activation sequences are activated in steps of 40–60 ms during imitation of facial expressions, corresponding to the 17–25 Hz frequency range (see Box 1 in Hari et al., 2010). Similarly, cortical sequences from 9 to 15 Hz follow finger imitation. On a behavioral level, changes in facial expressions (Perakula and Ruusuvuori, 2006), conversational turn taking (Stivers et al., 2009), and interpersonal coordination of finger tapping (Konvalinka et al., 2010) take place on the order of tens to hundreds of milliseconds. Therefore, as mutual interaction involves behavioral coupling between two people producing similar actions, and engages similar cognitive processes (such as predicting each other's actions, imitating each other's hand/finger movements, and jointly attending to joint actions) between interacting partners, it may not be so surprising that their brain rhythms are synchronized.

However, as this approach is very new and unexplored so far, there is not much previous literature to fall back on. We have mostly the “isolated brain” studies to dig up for explanations of described social processes, such as mirroring, mentalizing, and coordinating/cooperating. It thus becomes difficult to interpret what role these brain-to-brain couplings have in social interaction. We fall short of terms and concepts related to shared social phenomena, and begin to rely on literature on emergence as a source of explanation for what we are quantifying. Therefore, how these findings fit into the bigger picture of social cognition remains to be seen. In the following section, we consider some future steps in the two-brain approach, which may enable better understanding of social interaction at the level of mutually interacting brains.

## Consideration and future perspectives on two-brain approaches

Many of the two-brain studies have identified functional similarities between brains in interaction. This has been formulated in different ways—as information transfer, functional connectivity, causality, and/or phase synchronization. This implies one of two assumptions: (1) that brains of two interacting members are coupled via their behavior, or (2) that there is a brain-to-brain coupling mechanism between interacting partners that cannot be merely explained by the measured behavior of the two members. The first assumption does not necessarily require simultaneous brain recordings (or a mutual interaction), but does require that the brain activity of both members be recorded, which is synchronized in time to the behavioral input/output. The second assumption is difficult to explain given our lack of conceptual understanding of brain processes. The reductionist point of view, in search of a causal relationship, may be that this assumption postulates the existence of spurious brain-to-brain couplings. In other words, as one does not have access to another person's brain activity, it is difficult to understand how this coupling occurs. The non-reductionist perspective might be that these couplings are emergent—a result of complex interactions between the individual and the environment, and in turn, between an individual and the interacting partner, such that they cannot be reduced to the individual him/herself, or the controlled behavioral exchange measured between the participants. If this is indeed the phenomenon revealed, the resulting emergent properties of interaction require further quantification.

Quantitative and practical measures of autonomy and emergence have been discussed in a paper by Seth (2010). He proposes quantification via Granger-causality and Granger-autonomy, which together operationalize Granger-emergence, a measure of weak emergence. G-emergence, as he calls it, measures the degree to which an emergent process is simultaneously autonomous from and dependent on its causal constituents. For example, given micro variables x1 and x2, G-emergence of a macro variable x3 can be mathematically derived, such that it is both autonomous with respect to x1 and x2, and caused by x1 and x2. In the context of the two-brain approach, x1 and x2 may represent brain activity of interacting members 1 and 2, such that x3 is the emergent property of the interaction we are after (i.e., brain-to-brain coupling). This could be one potential future approach to the study of two-brain dynamics.

Other approaches to quantifying emergent brain dynamics of interacting partners have been proposed by Dumas (2011), in the context of information integration, and by Dodel et al. (2011), as dimensionality variation of brain dynamics. Information integration, analogous to the idea of emergence, is the amount of information produced by the whole of interacting elements, which is beyond the information produced by its parts (Tononi, 2008). This could be operationalized as a hyper-phase locking value (h-PLV), PLV between two interacting brains, which is proposed by Dumas to reflect dynamical sharing of information via inter-personal sensorimotor loops.

The Dodel et al. approach has not been implemented in other studies of interacting brains, to the best of our knowledge. It proposes use of dimensionality variation of joint brain dynamics to determine signatures of social coordination. Their method defines joint dynamics as an evolution along a particular manifold, which can be constructed both using behavioral and neural data of interacting members—in this case, by computing local subspaces of joint brain dynamics.

The above are potential future approaches to two-brain studies, which will continue to identify emergent phenomena of interactions, crucial to studies of real-time interactions. However, their prospects and outcomes are difficult to predict. We cannot escape from using terms such as shared representations and emergent interactive phenomena to define the desired outcome. More importantly, there remains a vast gap between the conceptual literature on social cognition as observation (i.e., in individual minds), and that of social cognition as interaction.

We propose another approach to interacting brains, using machine-learning methods to determine which signals emanating from each person are engaged during different forms of interaction. Machine-learning methods have been instrumental in the development of real-time decoding for brain-computer interfacing, and have previously been used in neuroimaging for the decoding of brain states of individuals (Haynes and Rees, 2006), and in the context of two-brain neuroimaging (Anders et al., 2011), among other applications (see Lemm et al., 2011 for an introduction). These analyses are useful for mining vast amounts of neural data, and particularly (in the context of this topic) for the decoding of relevant brain states, in order to distinguish them from uninformative signals.

The application of multivariate decoding models to two-brain data may allow partitioning of brain signals as belonging to distinct interactive conditions, for instance communication versus no communication. This method employs use of classifiers, which are functions that partition a set of objects into distinct classes, in order to identify which category a new observation belongs to, based on a training set of observations with a known category membership (Lemm et al., 2011). To provide an example, the technique could be used as follows: (a) design an experiment with two conditions, which are similar in their sensorimotor feedback, but different in the level of interaction—i.e., communication with another person, versus communication with a computer, (b) come up with a hypothesis regarding which temporal and/or spatial aspects of brain data relate to the studied communication, (c) partition the data concerning these features into the two classes (communication with person and communication with computer), (d) train a classifier on a subset of data from both conditions and across both members of the pair, (e) test the classifier (i.e., using N-fold cross-validation) employing feature selection to determine which brain signals from each member drive the classification, and finally, (f) test the resulting features/signals against the behavioral outcome (e.g., successful interactions, cooperation/competition, synchronized behavior).

With this technique, one may test which neural features (i.e., time/frequency components, sensors, and sources) successfully distinguish interactive from non-interactive conditions. Moreover, it enables us to see, statistically, which neural processes of each member are engaged during the interaction. In other words, while having a natural interaction between two people, which cannot be simulated via designated interactive roles, this approach aims at disentangling synchronized and/or complementary neural signals from the two brains that are engaged during different forms or degrees of interaction—i.e., one cooperates/other does not, one leads/other follows. This relates to previous studies that have found functional connectivity between the prefrontal areas of one person and parietal areas of the other predicting the cooperative strategy (Astolfi et al., 2010b). Similarly, one could use machine-learning techniques to address whether different combinations of signals from one and the other can predict different interactive strategies, without assuming a causal relation between the signals of the two brains.

It is important to note that this approach is not enactive (only behaviorally), as the analysis of neural interactions disentangles signals as belonging to member one and member two. However, it takes advantage of simultaneous recordings, given that it analyzes inter-personal neural processes during a naturalistic interaction. We believe that this application of multivariate decoding would be a useful approach for future analyses of two-brain studies.

The techniques mentioned in this section take advantage of the hyperscanning approach, by employing real-time interactions between people, while quantifying neural interactions between brains on a millisecond timescale—hence capturing both behavioral and neural adaptations inter-individually. They provide direct ways of combing different sets of data, and can allow us to study interactions between not just two brains, but three, four, or how ever many future research deems interesting to tackle.

## Conclusions

In this paper, we have reviewed the two-brain literature, which has explored neural dynamics and interactions between two interacting partners. The reviewed studies employing either fMRI, EEG, or NIRS recordings complement each other in quantifying hemodynamics or modulations of brain rhythms, both intra- and inter-personally, and integrate various conceptual frameworks. They have employed both representational and enactive approaches to social cognition via pseudo-interactive scenarios, turn-based interaction studies, and real-time mutual interactive studies. While reviewing the findings, approaches, and potential future methods, we also propose a pragmatic way to quantify two-brain interactions. In a well-defined paradigm with clear behavioral emergent markers of interaction, a multivariate decoding approach could combine brain data sets from two people, and use them to identify neural features particular to the studied interaction and the resultant role of each participant.

We believe that hyperscanning is necessary in future exploration of the underlying mechanisms of social interaction. It is the only way to tap into inter-brain processes, which we still know so little about. In interesting ways, the representational and enactive approaches may also point to a dual nature of social cognition. Social cognition as representation and social cognition as emergent patterns of interaction may point to mechanisms of *observing* and *participating* as two very different aspects social interaction. Understanding social cognition as participation seems to us to be the great challenge ahead, both at a behavioral and neuronal level. It accentuates the importance for cognition of the second person: the fact that so much of human consciousness and perception is directed against and mediated by inputs from other people (Roepstorff, 2001).

### Conflict of interest statement

The authors declare that the research was conducted in the absence of any commercial or financial relationships that could be construed as a potential conflict of interest.
